# The Hot Air Treatment

**Published:** 1900-09-01

**Authors:** 


### THE HOT AIR TREATMENT.

DR. DAVID WALSH writes: In your issue of the 25th you kindly gave a short abstract of an article of mine that appeared in the *Lancet* of August 18th. May I be allowed to point out that, in your otherwise excellent summary, no mention is made of the particular process on which the paper is based, namely, that invented by Mr. L. A. Tallerman?

